# Self-assembly of Ru_4_ and Ru_8_ assemblies by coordination using organometallic Ru(II)_2_ precursors: Synthesis, characterization and properties

**DOI:** 10.3762/bjoc.8.34

**Published:** 2012-02-28

**Authors:** Sankarasekaran Shanmugaraju, Dipak Samanta, Partha Sarathi Mukherjee

**Affiliations:** 1 Department of Inorganic and Physical Chemistry, Indian Institute of Science, Bangalore-560 012, India. Fax: 91-80-2360-1552; Tel: 91-80-2293-3352

**Keywords:** cages, macrocycles, ruthenium(II), self-assembly, self-sorting

## Abstract

Coordination-driven self-assembly of binuclear half-sandwich *p*-cymene ruthenium(II) complexes [Ru_2_(μ-η^4^-C_2_O_4_)(MeOH)_2_(η^6^-*p*-cymene)_2_](O_3_SCF_3_)_2_ (**1a**) or [Ru_2_(μ-η^4^-*N*,*N'*-diphenyloxamidato)(MeOH)_2_(η^6^-*p*-cymene)_2_](O_3_SCF_3_)_2_ (**1b**) separately with an imidazole-based tetratopic donor **L** in methanol affords two tetranuclear metallamacrocycles **2a** and **2b**, respectively. Conversely, the similar combination of **L** with 2,5-dihydroxy-1,4-benzoquinonato (dhbq) bridged binuclear complex [Ru_2_(μ-η^4^-C_6_H_2_O_4_)(MeOH)_2_(η^6^-*p*-cymene)_2_](O_3_SCF_3_)_2_ (**1c**) in 1:2 molar ratio resulted in an octanuclear macrocyclic cage **2c**. All the self-assembled macrocycles **2a**–**2c** were isolated as their triflate salts in high yields and were characterized fully by multinuclear (^1^H, ^13^C and ^19^F) NMR, infrared (IR) and electrospray ionization mass spectrometry (ESIMS). In addition, the molecular structure of macrocycle **2a** was established unequivocally by single-crystal X-ray diffraction analysis and adopts a tetranuclear rectangular geometry with the dimensions of 5.53 Å × 12.39 Å. Furthermore, the photo- and electrochemical properties of these newly synthesized assemblies have been studied by using UV–vis absorption and cyclic voltammetry analysis.

## Introduction

Self-assembly of metal-based molecular architectures through coordination has emerged as an active field of research as large numbers of intricate structural motifs can easily be derived in a single step from predesigned molecular building units. In the last two decades several interesting molecular architectures of defined shapes, sizes and functionality have been discovered with the aid of this powerful protocol [1–2]. The basic requirement for successful metal–ligand-directed self-assembly resides in the judicious designing of information-encoding molecular building blocks having complementary binding sites. Large numbers of molecular polygons and polyhedra have been synthesized mostly from *cis*-blocked Pd(II)- and Pt(II)-based building units due to their rigid square-planar coordination geometry as well as to their interesting photophysical properties [3–5]. However, we and others have recently reported several shape-persistent discrete macrocycles/cages using half-sandwich octanuclear Ru(II) piano-stool complexes in combination with polypyridyl or polycarboxylate donors [6–10]. Ligands with an imidazole functionality are of considerable interest due to the presence of imidazole in many biological systems, and also the better donor character of imidazole compared to widely used pyridyl donors. Owing to these properties, we were interested in incorporating an imidazole functionality into discrete molecules of defined shapes, and also to investigate their influence in terms of directionality in controlling the geometry of the resulting molecular architectures. Herein, we report the self-assembly reactions of [Ru_2_(μ-η^4^-C_2_O_4_)(MeOH)_2_(η^6^-*p*-cymene)_2_](O_3_SCF_3_)_2_ (**1a**) or [Ru_2_(μ-η^4^-*N,N'*-diphenyloxamidato)(MeOH)_2_(η^6^-*p*-cymene)_2_] (O_3_SCF_3_)_2_ (**1b**) and [Ru_2_(μ-η^4^-C_6_H_2_O_4_)(MeOH)_2_(η^6^-*p*-cymene)_2_](O_3_SCF_3_)_2_ (**1c**) with a tetratopic imidazole-based donor **L** in methanol at room temperature to generate two tetranuclear macrocycles of general formula [Ru_4_(μ-η^4^-C_2_O_4_ or *N*,*N'*-diphenyloxamidato)_2_(**L**)(η^6^-*p*-cymene)_4_](O_3_SCF_3_)_4_ (**2a**, **2b**) and an octanuclear macrocyclic cage [Ru_8_(μ-η^4^-C_6_H_2_O_4_)_4_(**L**)_2_(η^6^-*p*-cymene)_8_](O_3_SCF_3_)_8_ (**2c**), respectively in quantitative yields [**L** = 1,2,4,5-*tetrakis*(imidazol-1-yl)benzene]. The final assemblies were characterized by multinuclear (^1^H, ^13^C and ^19^F) NMR, IR and ESIMS analyses. The molecular structure of tetranuclear macrocycle **2a** was determined by single-crystal X-ray diffraction analysis, which reveals a tetranuclear geometry with the dimensions of 5.53 Å × 12.39 Å. In addition to their synthesis and characterization, the UV–vis absorption and cyclic voltammetry studies are reported.

## Results and Discussion

### Synthesis and characterization of the tetranuclear complexes

**Scheme 1:**
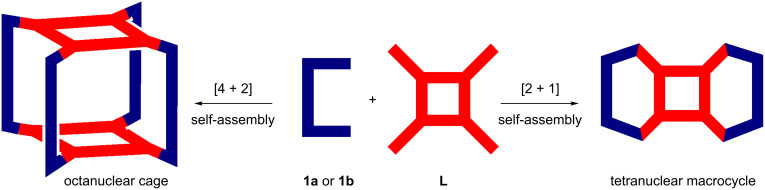
Possible octanuclear prism and tetranuclear macrocycle resulting from the combination of a tetradendate donor with a clip-type acceptor unit.

According to the “directional-bonding approach” and “symmetry-interaction model”, one can expect the formation of either a [4 + 2] assembled octanuclear tetragonal prism or a [2 + 1] assembled tetranuclear 2D structure upon reaction of a binuclear “clip”-type acceptor and a tetratopic donor (Scheme 1) [11].

**Scheme 2:**
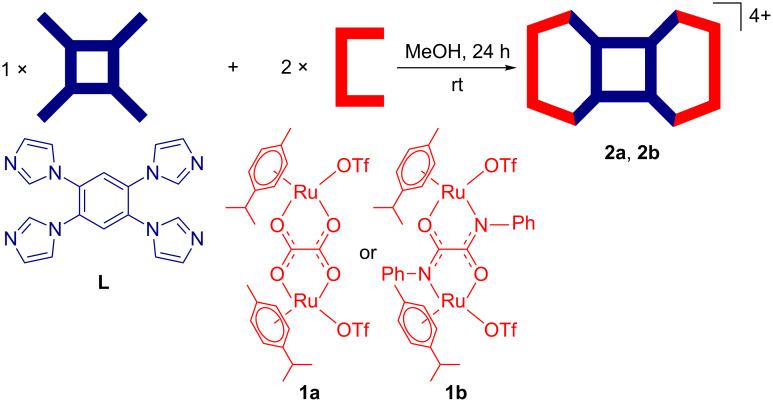
Formation of the tetranuclear complexes (**2a** and **2b**) upon the reaction of Ru(II)_2_ based acceptors **1a** or **1b** and tetraimidazole ligand **L** in methanol at room temperature.

Instead, the exclusive formation of two tetranuclear assemblies (**2a**, **2b**) upon mixing of binuclear Ru(II)_2_ acceptors **1a** or **1b** with an imidazole-based tetratopic donor **L** is interesting. The exclusive self-sorting of tetranuclear macrocycles (**2a** and **2b**) rather than the expected octanuclear molecular cage is, presumably, due to the perfect matching of the distance between two Ru(II) metal centers with the distance between the adjacent imidazole donor sites in **L**. Moreover, from an entropic point of view such [2 + 1] self-assembly is expected to be preferred over [4 + 2] assembly as the loss of entropy in the latter case is more. As shown in Scheme 2, the binuclear acceptor **1a** or **1b** was treated separately with an imidazole-based tetratopic donor **L** in a 2:1 molar ratio in methanol at room temperature to obtain [2 + 1] self-assembled macrocycles **2a** and **2b**, respectively, in high yields. The addition of solid **L** into a methanolic solution of the acceptor (**1a** or **1b**) lead to the immediate consumption of the solid and showed a sharp visible color change from light yellow to intense yellow. Both **2a** and **2b** are isolated as their triflate salts and are highly soluble in common organic solvents such as (CH_3_)_2_CO, CH_3_CN, CH_3_OH and CH_2_Cl_2_. The formation of the complexes (**2a** and **2b**) was initially characterized by IR, multinuclear (^1^H, ^13^C and ^19^F) NMR and ESIMS spectrometric analyses.

**Figure 1:**
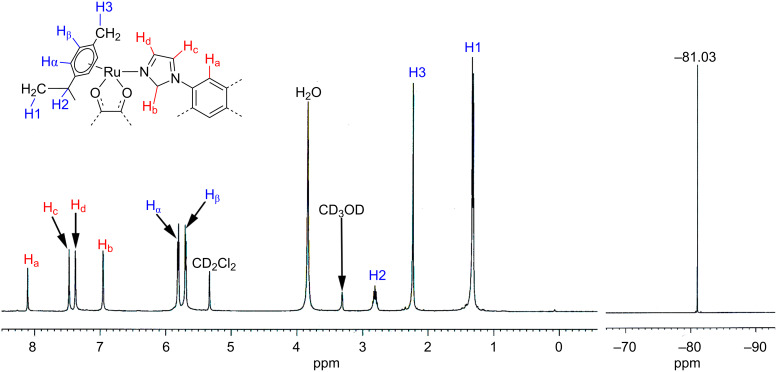
^1^H (left) and ^19^F (right) NMR spectra of tetranuclear macrocycle **2a** recorded in CD_2_Cl_2_–CD_3_OD with peak assignments.

The IR spectra (Figure S1, Supporting Information File 1) of the macrocycles showed strong bands at 1624.6 cm^−1^ (**2a**) and 1602 cm^−1^ (**2b**) corresponding to the symmetrical stretching frequencies (ν_CO_) of the carbonyl groups of the coordinated bis-bidentate oxalato (**2a**) or *N*,*N'*-diphenyloxamidato (**2b**) ligands. These bands, due to symmetrical stretching (ν_CO_) in the complexes, are slightly shifted to a higher energy region compared to those of the starting acceptors (**1a**, ν_CO_ = 1623.7 cm^−1^; **1b**, ν_CO_ = 1605.0 cm^−1^) due to the ligand-to-metal coordination. The appearance of a single peak in the ^19^F NMR spectrum at −81.03 ppm (**2a**) or −79.8 ppm (**2b**) indicated the presence of triflate counter anions in the same chemical environment in the resultant complexes (Figure 1 and Figure S3, Supporting Information File 1).

Four peaks correspond to the donor **L** in the aromatic region (δ 6.95–8.10 ppm) were found in the ^1^H NMR spectra of **2a** and **2b**. Protons corresponding to the capped *p*-cymene moiety appeared in the range of δ 5.14–5.81 ppm. Notably, the ^1^H signals of the protons of **L** exhibited a significant downfield shift compared to the unbound linker due to the loss of electron density upon ligand-to-metal coordination (Figure 1 and Figure S2, Supporting Information File 1). The high solubility of the assemblies in common organic solvents and the symmetric nature of ^1^H NMR spectra primarily ruled out the formation of a polymer with extended coordination. Although the initial characterization of the self-assembled species by NMR spectroscopy intimated the ligand-to-metal coordination, it does not provide any information about the exact composition and nuclearity of the final products.

**Figure 2:**
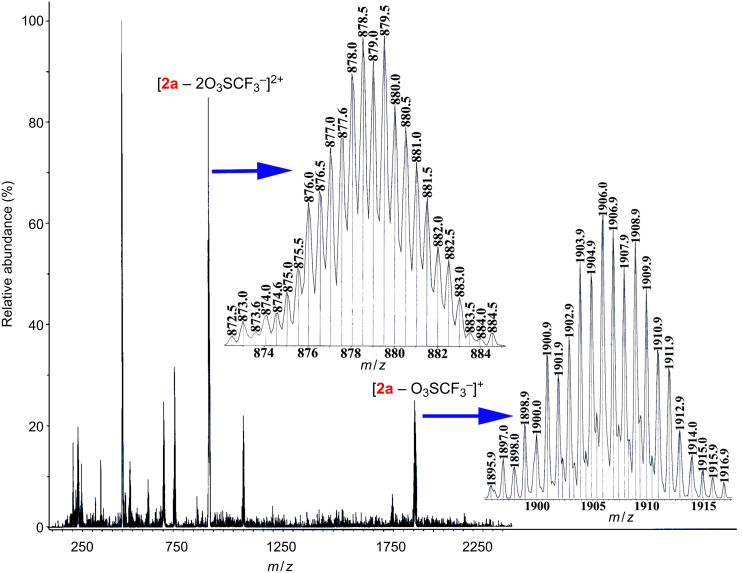
ESIMS spectrum of the macrocycle **2a** recorded in acetonitrile. Inset: Experimentally observed isotopic distribution for [**2a** − O_3_SCF_3_^−^]^+^ and [**2a** − 2O_3_SCF_3_^−^]^2+^ fragments.

Electrospray ionization mass spectrometry (ESIMS) can be used as a soft-ionization method to elucidate the exact composition of the product [12]. The ESIMS analysis confirmed the formation of the rather unexpected [2 + 1] self-assembled tetranuclear macrocycles **2a** and **2b** by the appearance of multiply charged fragmented ions (Figure 2 and Figure S4, Supporting Information File 1). The multiply charged ions for **2a** at *m*/*z* = 1906.9 [**2a** − O_3_SCF_3_^−^]^+^, 879.0 [**2a** − 2O_3_SCF_3_^−^]^2+^; for **2b** at *m*/*z* = 1029.53 [**2b** − 2O_3_SCF_3_^−^]^2+^, 637.27 [**2b** − 3O_3_SCF_3_^−^]^3+^ were observed and these peaks were well resolved isotopically (Figure 2 and Figure S4, Supporting Information File 1). The appearance of the expected peaks along with the isotopic patterns was in strong support of the formation of [2 + 1] self-assembled products in both cases.

**Figure 3:**
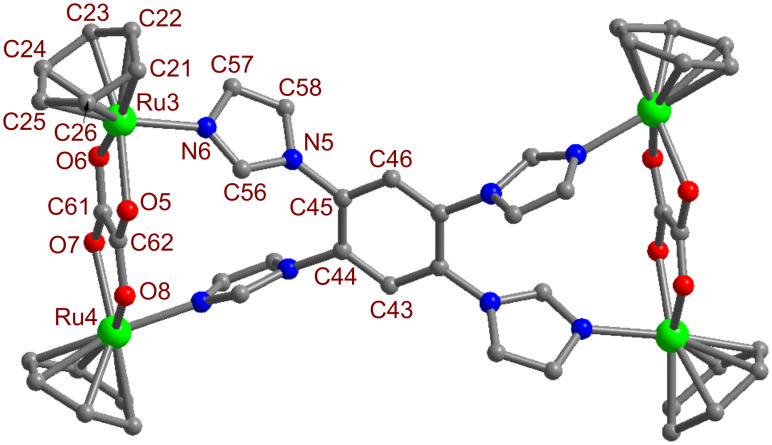
A ball and stick representation of **2a** with atom numbering. Color code: Ru = green; O = red; N = blue; C = dark gray. All hydrogen atoms, triflate counter anions, isopropyl and methyl groups of the *p*-cymene moiety are omitted for the sake of clarity.

Finally, the molecular structure of the tetranuclear metallamacrocycle [(*p*-cymene)_4_Ru_4_(μ-η^4^-oxalato)_2_(**L**)]^4+^(O_3_SCF_3_^−^)_4_ (**2a**) was unambiguously determined by single-crystal X-ray diffraction analysis. Single crystals of **2a** of high enough quality for X-ray diffraction were grown by slow vapor diffusion of diethyl ether into a methanolic solution of **2a** at room temperature. Macrocycle **2a** was crystallized in a tetragonal crystal system with *I*41/*a* space group having sixteen formula units per unit cell. A perspective view of the macrocycle **2a** is depicted in Figure 3 with atom numbering, and its selected bond parameters are summarized in Table S1, Supporting Information File 1.

The crystal-structure analysis of **2a** shows a tetranuclear geometry with dimensions of 5.53 Å × 12.39 Å, and the coordination geometry around each Ru(II) is octahedral. Capped *p*-cymene occupies three coordinating sites of each Ru(II). The bridging dianionic oxalato (C_2_O_4_^2−^) ligand binds through its two oxygen atoms. The imidazole nitrogen of the donor **L** occupies the sixth coordination site. The oxalato ligand connects to two ruthenium metal centers through four oxygen atoms. The average Ru–N and Ru–O bond distances are 2.140 Å and 2.141 Å, respectively. The four donor sites of **L** are coordinated to four different Ru(II) metal centers and thereby it adopts a tetranuclear rectangular geometry. The solid-state packing of macrocycle **2a** along the crystallographic *c*-axis results in a highly porous structure with square-type channels due to the π–π interactions between the adjacent macrocycles (Figure S8, Supporting Information File 1). Triflate (O_3_SCF_3_^−^) counter anions are located outside of the porous channel (Figure S8, Supporting Information File 1).

### Synthesis and characterization of the octanuclear cage

**Scheme 3:**
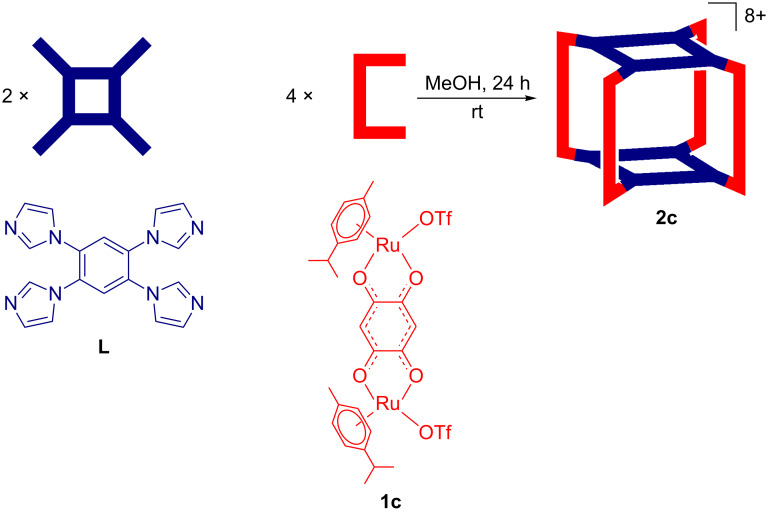
Formation of an octanuclear macrocycle **2c** upon reaction of Ru(II)_2_-based acceptors **1c** with imidazole-based tetratopic donor **L** in methanol at room temperature.

The construction of an octanuclear macrocyclic cage **2c** was accomplished following a similar procedure as adopted for the synthesis of tetranuclear rectangular complexes **2a** and **2b**. Drop-wise mixing of a methanolic solution of binuclear acceptor **1c** into a suspension of tetradendate linker **L** in methanol in 2:1 molar ratio afforded the exclusive formation of the [4 + 2] self-assembled macrocyclic cage **2c** in good yield after 24 h of stirring at room temperature (Scheme 3). The obvious reason for the formation of an octanuclear macrocyclic cage **2c** in contrast to the tetranuclear rectangles **2a** and **2b** is attributed to the increased length of the acceptor unit **1c** compared to **1a** or **1b**. The immediate consumption of the suspended donor **L** in the clear solution and the observable color change from purple to deep red indicated the progress of the self-assembly reaction. The initial characterization of the isolated macrocycle by multinuclear (^1^H, ^13^C and ^19^F) NMR suggested the formation of a single and symmetric macrocyclic complex.

**Figure 4:**
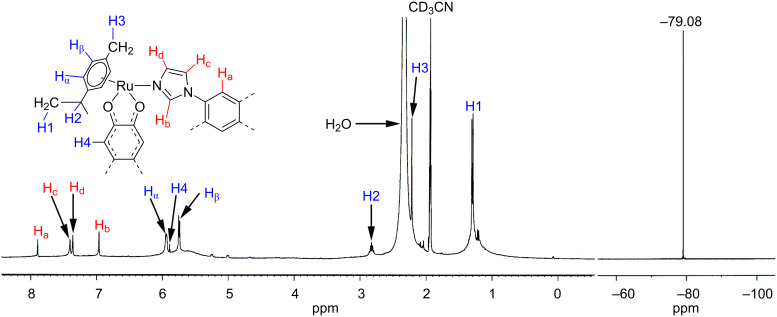
^1^H (left) and ^19^F (right) NMR spectrum of the macrocycle **2c** recorded in CD_3_CN with the peak assignments.

The infrared spectra (Figure S1, Supporting Information File 1) of the macrocycle (**2c**) showed a strong band at 1521.2 cm^−1^ corresponding to the symmetric stretching frequency (ν_CO_) of the carbonyl groups of the coordinated bis-bidentate quinonato ligand, and this symmetric stretching frequency (ν_CO_) in the isolated macrocycle is slightly shifted to the higher energy region compared to that of the starting acceptor (**1c**, ν_CO_ = 1515.8 cm^−1^) due to the ligand-to-metal coordination. The ^1^H NMR spectra of the macrocycle **2c** established the expected peaks in the aromatic region corresponding to the tetratopic donor **L** and the proton resonance of capped *p*-cymene ligand. Moreover, the peak of **L** in the macrocyclic complex exhibits a significant downfield shift due to the ligand–metal coordination (Figure 4). The assignment of the proton signals in the ^1^H NMR spectra was confirmed by the ^1^H–^1^H COSY NMR (Figure S5, Supporting Information) spectral analysis. Furthermore, the appearance of prominent peaks in the ESIMS spectra of the multiply charged ions for **2c** at *m*/*z* = 2008.0 [**2c** − 2O_3_SCF_3_^−^]^2+^, 1288.0 [**2c** − 3O_3_SCF_3_^−^]^3+^, 928.9 [**2c** − 4O_3_SCF_3_^−^]^4+^ indicated the formation of a [4 + 2] self-assembled octanuclear macrocyclic cage. The peaks corresponding to the [**2c** − 2O_3_SCF_3_^−^]^2+^ and [**2c** − 3O_3_SCF_3_^−^]^3+^ fragments are well resolved isotopically (Figure S6, Supporting Information File 1).

**Figure 5:**
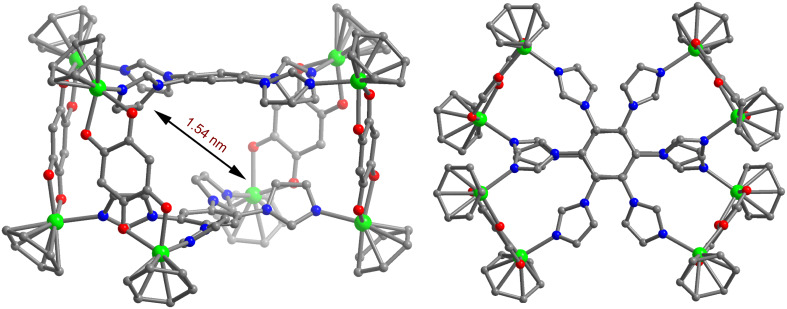
Side (left) and top (right) view of the energy-minimized structures of the octanuclear macrocycle **2c**. Color code: green = Ru, blue = N, red = O, gray = C. The hydrogen atoms, methyl and isopropyl group of the cymene moiety have been removed for the sake of clarity.

Unfortunately, all efforts to obtain diffraction-quality single crystals of the macrocycle **2c** have so far been unsuccessful. However, the analysis of multinuclear NMR (^1^H, ^13^C and ^19^F) in concurrence with the ESIMS study is fully consistent with the formation of a [4 + 2] self-assembled octanuclear metallacage. In view toward gaining further insight into the structural characteristics of the newly designed macrocycle, the energy-minimized structure of the macrocycle **2c** was obtained by means of molecular mechanics universal-force-field simulation (MMUFF) [13]. A perspective view of the energy-minimized structure of the macrocycle **2c** is depicted in Figure 5. The optimized structures of the macrocycle **2c** suggested the formation of an octanuclear macrocycle having an internal diameter of 1.54 nm. Notably, the quinonato-bridged ruthenium clips are tilted out of the plane of the imidazole donor in **2c** in order to minimize the steric influence between the acceptor clips. Therefore, macrocycle **2c** adopts a staggered conformation as was observed in similar types of 3D cages [14].

### UV–vis absorption and electrochemical studies

One of the strongest driving factors to design multifarious functional supramolecular complexes is attributed to their interesting functional properties. In general, the utilization of functional properties would be realized more from macrocycles containing transition metals, due to their high sensitivity towards various external stimuli, compared to purely organic macrocycles. In this regard, substantial efforts have been devoted to the design of novel, nanoscopic and functional metallamacrocycles and also to the study of their functional properties [15]. The photo- and electrochemical properties of our newly synthesized macrocycles (**2a** and **2b**) are studied by UV–vis absorption and cyclic voltammetry investigations and the obtained results are summarized in Table 1.

**Table 1:** Results of UV–vis absorption (in CH_3_CN, 1.0 × 10^−5^ M) and electrochemical (in CH_2_Cl_2_, 0.1 M *n*-Bu_4_NPF_6_) studies of macrocycles **2a**–**2c** at 298 K.

Macrocycle	Absorption maxima^a^ λ_max_ (nm)	Molar extinction coefficient ε × 10^3^ M^−1^cm^−1^ λ_max_ (nm)	*E*_1/2_ (*V* vs SCE)

**2a**	**309**, 383	23 (309)	–
**2b**	**328**	58 (328)	−0.15
**2c**	**293**, 499	63 (293)	−0.08, −0.14, −0.12

^a^Values in bold represent the highest absorption (λ_max_) maxima.

**Figure 6:**
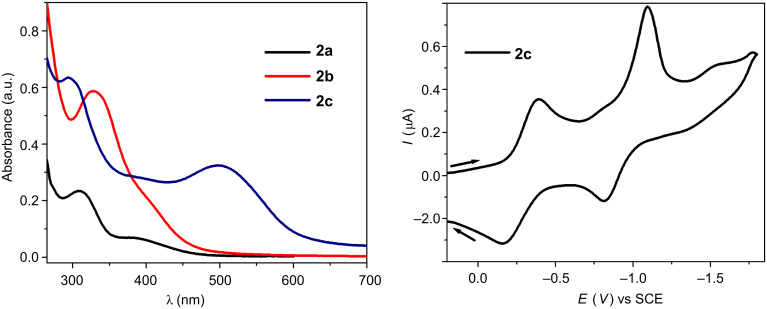
UV–vis absorption spectrum (left) of macrocycles (**2a**–**2c**) recorded in CH_3_CN at 298 K, and cyclic voltammogram of **2c** (right) performed in CH_2_Cl_2_/0.1 M (*n*-Bu)_4_NPF_6_ with a scan rate of 100 mV s^−1^ versus SCE at 298 K.

The UV–vis absorption spectra were recorded from a CH_3_CN solution (1.0 × 10^−5^ M) of the macrocycles at room temperature. The electronic absorption spectra (Figure 6) of the macrocycles **2a**–**2c** exhibit intense bands at λ_max_ (ε) 309 nm (2.3 × 10^4^ M^−1^ cm^−1^), 383 nm (6.8 × 10^3^ M^−1^ cm^−1^) for **2a**; λ_max_ (ε) = 328 nm (5.8 × 10^4^ M^−1^ cm^−1^) for **2b** and λ_max_ (ε) = 293 nm (6.3 × 10^4^ M^−1^ cm^−1^), 499 nm (3.2 × 10^4^ M^−1^ cm^−1^) for **2c**. The peaks in the ranges of 293–309 nm and 328–499 nm can be ascribed to both intra- and intermolecular π–π* and metal-to-ligand charge-transfer transitions associated with the capped *p*-cymene–ruthenium moiety, respectively. The electrochemical behavior of the macrocycles (**2a**–**2c**) was examined by using a Pt-disk electrode in dichloromethane with 0.1 M *n*-Bu_4_NPF_6_ as the supporting electrolyte and at 100 mV s^−1^ scan rate. The obtained redox responses of the macrocycles were found to be entirely different. Macrocycle **2c** shows three well-anchored quasi-reversible reductions at −0.39, −1.09 and −1.54 V and their corresponding anodic peak current at −0.16, −0.81 and −1.38 V (Figure 6). On the other hand, complex **2b** showed a single quasi-reversible redox response of the cathodic peak current at −1.53 V and the anodic peak counterpart potential approximately at −1.24 V (Figure S7, Supporting Information File 1). Surprisingly, macrocycle **2a** showed no anodic or cathodic peak current intensity within the range of +0.2 to −0.2 V, which implies the electrochemical inertness of **2a** (Figure S7, Supporting Information File 1). The observed anodic and cathodic peak currents of **2c** are almost equal, indicating the high chemical stability on the time scale of the voltammetry experiments. Notably, the cathodic peak current intensity of **2b** is much higher than its anodic peak current, and this can be attributed to the deposition of macrocycles on the electrode surface. Based on the electrochemical activity of other reported ruthenium complexes, the observed cathodic peaks are roughly assigned to one-electron reductions of the bridged quinonato or diphenyloxamidato ligands, and the oxidation is attributed to the Ru(II)/Ru(III) redox couples [16–17].

## Conclusion

In conclusion, we synthesized two new tetranuclear macrocycles **2a**, **2b** and an octanuclear molecular prism **2c**, by coordination-driven self-assembly of an imidazole-based tetratopic donor linker **L** in combination with Ru(II)-based binuclear “clip” acceptors. All three of the self-assembled macrocycles were fully characterized by multinuclear (^1^H, ^13^C and ^19^F) NMR, IR and ESIMS spectroscopic studies. In addition, the formation of a tetranuclear structure of **2a** was unambiguously established by analysis of single-crystal X-ray diffraction data. Though the formation of discrete structures from pyridyl donors has been well established, the quantitative formation of **2a**, **2b** and **2c** from an imidazole-type donor is new and suggests the feasibility of using imidazole derivatives as potential building units. The lengths of **1a** and **1b** fit well with the distance between two imidazole units in **L** to form the entropically favored [2 + 1] self-assembled structures **2a** and **2b**, respectively. As the distance between the two Ru(II) in **1c** is greater compared to the distance between the neighboring imidazole units in **L**, a similar reaction with **1c** allowed the formation of the 3D cage **2c**. The use of imidazole-based donor linkers in combination with organometallic half-sandwich ruthenium(II) precursors may generate a wide variety of complex molecular architectures with interesting functional properties.

## Experimental

### Materials and methods

The acceptor clips [Ru_2_(μ-η^4^-C_2_O_4_)(MeOH)_2_(η^6^-*p*-cymene)_2_](O_3_SCF_3_)_2_ (**1a**), [Ru_2_(μ-η^4^-*N*,*N'*-diphenyloxamidato)(MeOH)_2_(η^6^-*p*-cymene)_2_](O_3_SCF_3_)_2_ (**1b**) and [Ru_2_(μ-η^4^-C_6_H_2_O_4_)(MeOH)_2_(η^6^-*p*-cymene)_2_](O_3_SCF_3_)_2_ (**1c**) were synthesized under a dry nitrogen atmosphere by means of a standard Schlenk technique following the reported procedures [18–20]. The solvents were dried and distilled according to the standard literature procedures. 1,2,4,5-tetrabromobenzene and α-phellandrene were purchased from commercial sources and used without further purification. 1,2,4,5-Tetrakis(imidazol-1-yl)benzene (**L**) was synthesized by following the reported procedure [21]. NMR spectra were recorded on a Bruker 400 MHz spectrometer. The chemical shifts (δ) in the ^1^H NMR spectra are reported in ppm relative to tetramethylsilane (Me_4_Si) as the internal standard (0.0 ppm) or the proton resonance resulting from incomplete deuteration of the NMR solvents: CD_3_CN (1.94), CD_3_OD (3.33) and CD_2_Cl_2_ (5.33). ^19^F NMR were recorded at 376.5 MHz and the chemical shifts (δ) are reported in ppm relative to the external standard Cl_3_CF (0.00). ESIMS experiments were performed on a Bruker Daltonics (Esquire 300 Plus ESI model) with standard spectroscopic grade solvents CH_3_CN or CH_3_OH. IR spectra were recorded on a Bruker ALPHA FT-IR spectrometer. Electronic absorption studies were carried out on a Perkin Elmer LAMBDA 750 UV–vis spectrophotometer. The electrochemical measurements were performed in a three-electrode system consisting of a platinum electrode, a glassy-carbon counter electrode and a standard calomel reference electrode. All the potentials of the macrocycles (1.0 × 10^−3^ M in DCM) were measured with 0.1 M *n*-Bu_4_NPF_6_ as the supporting electrolyte at room temperature with a scan rate of 100 mV s^−1^.

**General procedure for the synthesis of 2a–2c:** To a solid form of the tetratopic donor **L** was added separately a clear solution of the binuclear acceptor clip (**1a**–**1c**) in methanol (4 mL) in 1:2 molar ratio, and the mixture was stirred at room temperature for 24 h in a closed 4 mL glass vial. Immediate consumption of the suspended tetratopic donor (**L**) and significant visual color changes from light yellow to intense yellow for **2a** and **2b** or purple to deep red for **2c** evidenced the progress of the reactions. The solvent was removed under vacuum and the residue was dissolved in the minimum amount of dichloromethane (DCM) (~2 mL). Diethyl ether was mixed with concentrated clear solutions to precipitate out the expected macrocycles in pure form.

**2a:** Acceptor clip **1a** (29.5 mg, 0.032 mmol) and tetratopic donor **L** (5.5 mg, 0.016 mmol) were stirred in methanol (4 mL) to obtain **2a**. Isolated yield: 85%. ^1^H NMR (400 MHz, CD_2_Cl_2_/CD_3_OD) δ 8.10 (s, 2H, H_a_-phenyl), 7.47 (s, 4H, H_c_-imidazole), 7.37 (s, 4H, H_d_-imidazole), 6.95 (s, 2H, H_b_-imidazole), 5.81 (d, *J* = 6.0 Hz, 8H, H_α_-cymene), 5.70 (d, *J* = 6.0 Hz, 8H, H_β_-cymene), 2.84–2.77 (septet, 4H, H2-cymene), 2.23 (s, 12H, H3-cymene), 1.33 (d, 24H, H1-cymene); ^19^F NMR (376.5 MHz, CD_2_Cl_2_/CD_3_OD) δ −81.03 ppm; ^13^C NMR (100 MHz, CD_3_COCD_3_) δ 171.65, 140.12, 136.08, 131.62, 129.14, 126.36, 124.65, 101.55, 98.10, 82.85, 81.12, 54.51, 31.56, 22.03, 17.79; IR (neat) ν (cm^−1^): 3128.8 (w, C=C of aromatic), 1624.6 (s, CO-oxalate), 1254.9 (s, C–F of CF_3_); ESIMS (*m*/*z*): 1907.16 [**2a** − O_3_SCF_3_^−^]^+^, 879.0 [**2a** − 2O_3_SCF_3_^−^]^2+^; UV–vis (1.0 × 10^−5^ M, CH_3_CN) λ_max_ nm (ε): 309 (2.3 × 10^4^ M^−1^ cm^−1^), 383 (6.8 × 10^3^ M^−1^ cm^−1^); Anal. calcd for C_66_H_70_F_12_N_8_O_20_Ru_4_S_4_: C, 38.56; H, 3.43; N, 5.45; found: C, 38.84; H, 3.68; N, 5.71.

**2b:** Acceptor clip **1b** (34.3 mg, 0.032 mmol) and tetratopic donor **L** (5.5 mg, 0.016 mmol) were stirred in methanol (4 mL) to obtain **2b**. Isolated yield: 88%. ^1^H NMR (400 MHz, CD_3_OD) δ 8.19 (s, 4H, H_c_-imidazole), 8.17 (s, 4H, H_d_-imidazole), 7.82 (s, 4H, H_b_-imidazole), 7.46–7.21 (m, 20H, *N*-phenyl), 7.17 (s, 2H, H_a_-imidazole), 5.63–5.14 (m, 16H, H_α,β_-cymene), 2.50–2.48 (septet, 4H, H2-cymene), 1.71 (s, 12H, H3-cymene), 1.12 (d, 24H, H1-cymene); ^19^F NMR (376.5 MHz, CD_3_OD) δ −79.84; ^13^C NMR (100 MHz, CD_3_NO_2_) δ 170.89, 147.02, 139.76, 135.54, 132.74, 129.87, 128.63, 127.03, 125.45, 123.82, 123.00, 119.81, 104.32, 99.43, 85.27, 83.99, 82.23, 81.35, 65.70, 31.47, 22.30, 20.99, 17.16, 14.70; IR (neat) ν (cm^−1^): 3136 (w, C=C of aromatic), 1602 (s, CO-oxamide), 1262 (s, C–F of CF_3_); ESIMS (*m*/*z*): 1029.53 [**2b** − 2O_3_SCF_3_^−^]^2+^, 637.27 [**2b** − 3O_3_SCF_3_^−^]^3+^; UV–vis (1.0 × 10^−5^ M, CH_3_CN) λ_max_ nm (ε): 328 (5.8 × 10^4^ M^−1^ cm^−1^); Anal. calcd for C_90_H_90_F_12_N_12_O_16_Ru_4_S_4_: C, 45.88; H, 3.85; N, 7.13; found: C, 46.59; H, 4.17; N, 7.43;

**2c:** Acceptor clip **1c** (31.1 mg, 0.032 mmol) and tetratopic donor **L** (5.5 mg, 0.016 mmol) were stirred in methanol (4 mL) to obtain **2c**. Isolated yield: 80%.^1^H NMR (400 MHz, CD_3_CN) δ 7.89 (s, 4H, H_a_-phenyl), 7.40 (s, 8H, H_c_-imidazole), 7.36 (s, 8H, H_d_-imidazole), 6.96 (s, 8H, H_b_-imidazole), 5.94 (d, *J* = 5.6 Hz, 16H, H_α_-cymene), 5.88 (s, 4H, H4-quinone), 5.75 (d, *J* = 6.0 Hz, 16H, H_β_-cymene), 2.86–2.79 (septet, 8H, H2-cymene), 2.22 (s, 24H, H3-cymene), 1.31 (d, 48H, H^1^-cymene); ^19^F NMR (376.5 MHz, CD_3_CN) δ −79.08; ^13^C NMR (100 MHz, CD_3_NO_2_) δ 183.92, 140.62, 134.75, 131.45, 124.09, 122.92, 119.74, 103.76, 99.10, 84.88, 83.65, 79.47, 78.00, 31.78, 22.27, 17.70; IR (neat) ν (cm^−1^): 3128.8 (w, C=C of aromatic), 1521.2 (s, CO-oxalate), 1252.1 (s, C–F of CF_3_); ESIMS (*m*/*z*): 2008.0 [**2c** − 2O_3_SCF_3_^−^]^2+^, 1288.0 [**2c** − 3O_3_SCF_3_^−^]^3+^, 928.9 [**2c** − 4O_3_SCF_3_^−^]^4+^; UV–vis (1.0 × 10^−5^ M, CH_3_CN) λ_max_ nm (ε): 293 (6.3 × 10^4^ M^−1^ cm^−1^), 499 (3.2 × 10^4^ M^−1^ cm^−1^); Anal. calcd for C_148_H_148_F_24_N_16_O_40_Ru_8_S_8_: C, 41.23; H, 3.46; N, 5.20; found: C, 41.83; H, 4.16; N, 5.60.

**X-ray data collection and structure refinements:** Crystallographic data for **2a** were collected on a Bruker SMART APEX CCD diffractometer with the SMART/SAINT software [22]. X-ray-quality crystals were mounted on a glass fiber with traces of viscous oil. Intensity data were collected by using graphite-monochromatized Mo Kα radiation (0.7107 Å) at 150 K. The structures were solved by direct methods using SHELX-97 incorporated in WinGX [23–27]. Empirical absorption corrections were applied with SADABS [25]. All nonhydrogen atoms were refined with anisotropic displacement coefficients. Hydrogen atoms were assigned isotropic displacement coefficients, *U*(H) = 1.2*U*(C) or 1.5*U* (C-methyl), and their coordinates were allowed to ride on their respective carbons.

CCDC-845980 (for complex **2a**) contains the supplementary crystallographic data reported in this paper. The data can be obtained free of charge from the Cambridge Crystallographic Data Center via http://www.ccdc.cam.ac.uk/data_request/cif.

**Crystallographic data:** C_66_H_70_F_12_N_8_O_20_Ru_4_S_4_, *M*_r_ = 2055.86, tetragonal, space group *I*41/*a*, *a* = 27.975(5) Å, *b* = 27.975(5) Å, *c* = 48.786(17) Å, α = 90°, β = 90°, γ = 90°, *V* = 38179(16) Å^3^, *Z* = 16, ρ_calcd._ = 1.431 g cm^−1^, Mo Kα radiation (graphite monochromatic, λ = 0.71073 Å), *T* = 150 K, final R indices [I > 2σ(I)]: *R*_1_ = 0.1152 (5366), *wR*_2_ = 0.4337 (18769).

## Supporting Information

File 1 Infrared and NMR spectra of the macrocycles **2a**, **2b** and **2c** and solid-state packing diagram of macrocycle **2a**.

File 2 Crystallographic details of **2a**.

## References

[1] Northrop B H, Yang H-B, Stang P J (2008). Chem Commun.

[2] Yoshizawa M, Klosterman J K, Fujita M (2009). Angew Chem, Int Ed.

[3] Chakrabarty R, Mukherjee P S, Stang P J (2011). Chem Rev.

[4] Shanmugaraju S, Bar A K, Chi K-W, Mukherjee P S (2010). Organometallics.

[5] Shanmugaraju S, Joshi S A, Mukherjee P S (2011). Inorg Chem.

[6] Therrien B (2009). Eur J Inorg Chem.

[7] Han Y-F, Jia W-G, Yu W-B, Jin G-X (2009). Chem Soc Rev.

[8] Wang M, Vajpayee V, Shanmugaraju S, Zheng Y-R, Zhao Z, Kim H, Mukherjee P S, Chi K-W, Stang P J (2011). Inorg Chem.

[9] Shanmugaraju S, Bar A K, Mukherjee P S (2010). Inorg Chem.

[10] Shanmugaraju S, Bar A K, Joshi S A, Patil Y P, Mukherjee P S (2011). Organometallics.

[11] Northrop B H, Chercka D, Stang P J (2008). Tetrahedron.

[12] Fujita M, Yu S-Y, Kusukawa T, Funaki H, Ogura K, Yamaguchi K (1998). Angew Chem, Int Ed.

[13] ArgusLab 4.0.

[14] Govindaswamy P, Linder D, Lacour J, Süss-Fink G, Therrien B (2006). Chem Commun.

[15] Amijs C H M, van Klink G P M, van Koten G (2006). Dalton Trans.

[16] Mattsson J, Govindaswamy P, Renfrew A K, Dyson P J, Stěpnička P, Süss-Fink G, Therrien B (2009). Organometallics.

[17] Kitagawa S, Kawata S (2002). Coord Chem Rev.

[18] Barry N P E, Govindaswamy P, Furrer J, Süss-Fink G, Therrien B (2008). Inorg Chem Commun.

[19] Zhang W-Z, Han Y-F, Lin Y-J, Jin G-X (2009). Dalton Trans.

[20] Yan H, Süss-Fink G, Neels A, Stoeckli-Evans H (1997). J Chem Soc, Dalton Trans.

[21] Rit A, Pape T, Hepp A, Hahn F E (2011). Organometallics.

[22] (2004). SMART/SAINT.

[R23] 23Sheldrick, G. M. *SHELX-97,* Program for the Solution and Refinement of Crystal Structures; University of Göttingen: Göttingen, Germany, 1998.

[24] (2003). WinGX: An Integrated System of Windows Programs for the Solution, Refinement; Analysis for Single Crystal X-ray Diffraction Data.

[R25] 25Sheldrick, G. M. *SADABS,* Bruker Nonius Area Detector Scaling and Absorption Correction; Version 2.05; University of Göttingen: Göttingen, Germany, 1999.

[R26] 26ORTEP-3 for Windows, Version, 1.08.Farrugia, L. J. *J. Appl. Crystallogr.* **1997,** *30,* 565. doi:10.1107/S0021889897003117

[27] Spek A L (1990). Acta Crystallogr.

