# Supplementary material for: Self-assembly of Ru4 and Ru8 assemblies by coordination using organometallic Ru(II)2 precursors: Synthesis, characterization and properties
Source: Beilstein J Org Chem. 2012 Feb 28;8:313–22. doi: 10.3762/bjoc.8.34 (PMC3302095; doi:10.3762/bjoc.8.34)
Supplement: File 1 — Infrared and NMR spectra of the macrocycles 2a, 2b and 2c and solid-state packing diagram of macrocycle 2a. [file Beilstein_J_Org_Chem-08-313-s001.pdf]

Supporting Information

for

Self-assembly of Ru_4 and Ru_8 assemblies by coordination using organometallic $Ru(II)_2$ precursors: Synthesis, characterization and properties

Sankarasekaran Shanmugaraju, Dipak Samanta and Partha Sarathi Mukherjee*

Address: Department of Inorganic and Physical Chemistry, Indian Institute of Science, Bangalore-560 012, India. Fax: 91-80-2360-1552; Tel: 91-80-2293-3352

E-mail: Partha Sarathi Mukherjee* - psm@ipc.iisc.ernet.in

*Corresponding author

Infrared and NMR spectra of the macrocycles 2a, 2b and 2c and solid-state packing diagram of macrocycle 2a.

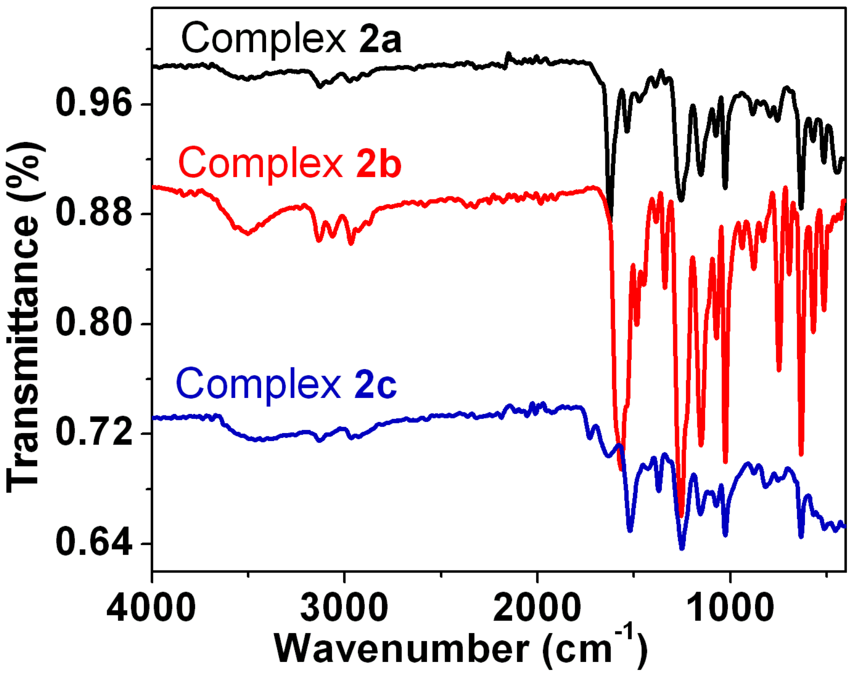

Figure S1: Infrared spectrum of the macrocycles **2a**–**2c**.

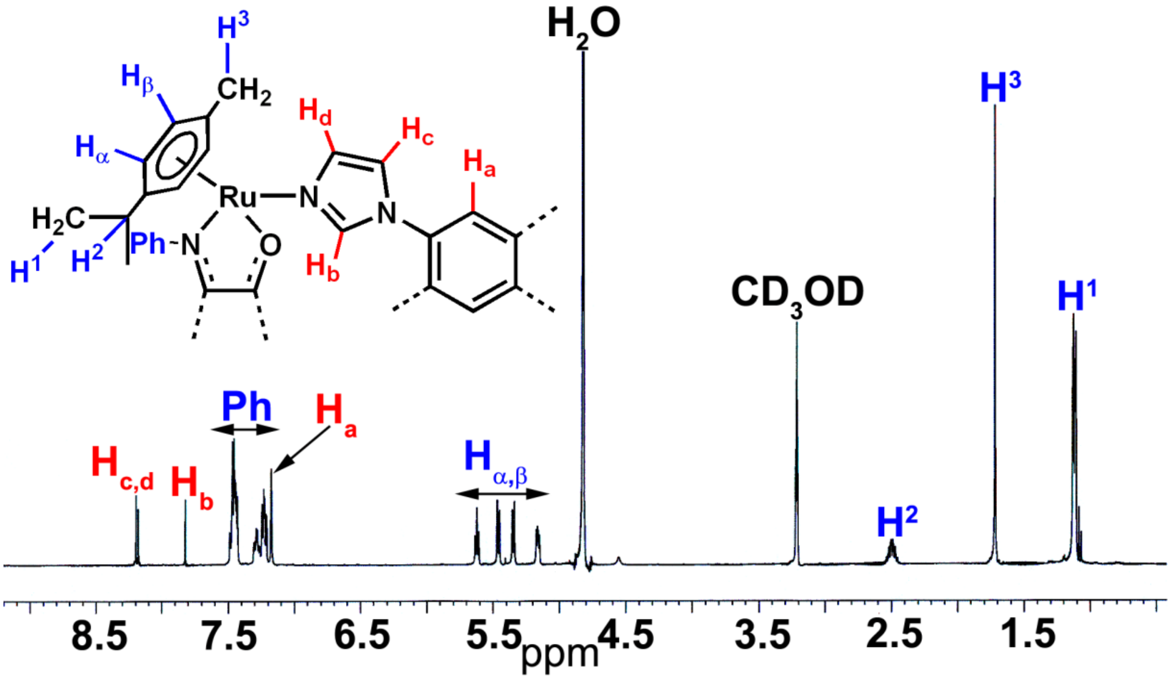

Figure S2: ^{1}H NMR spectrum of the macrocycle **2b** recorded in CD_3OD with the peak assignments.

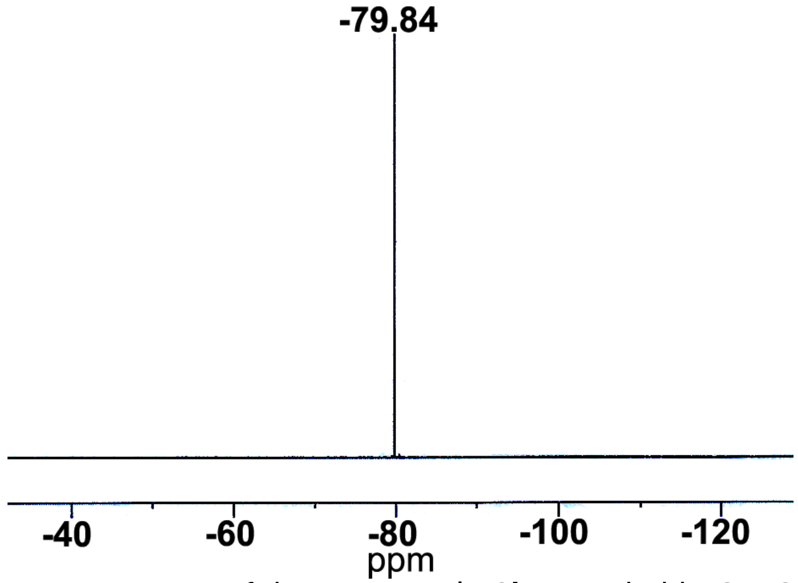

Figure S3: ^{19}F NMR spectrum of the macrocycle **2b** recorded in CD_3OD with the peak assignment.

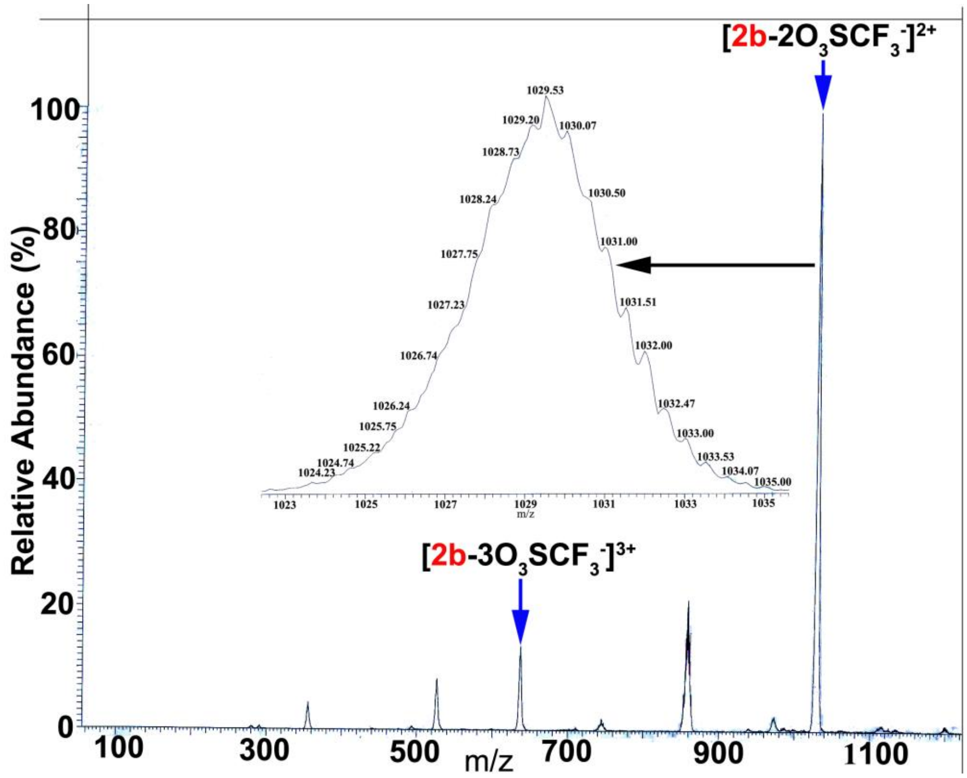

Figure S4: ESIMS spectrum of the macrocycle **2b** recorded in acetonitrile. Inset: experimentally observed isotopic distribution for the [**2b** – $2O_3SCF_3^-$]$^{2+}$ fragment.

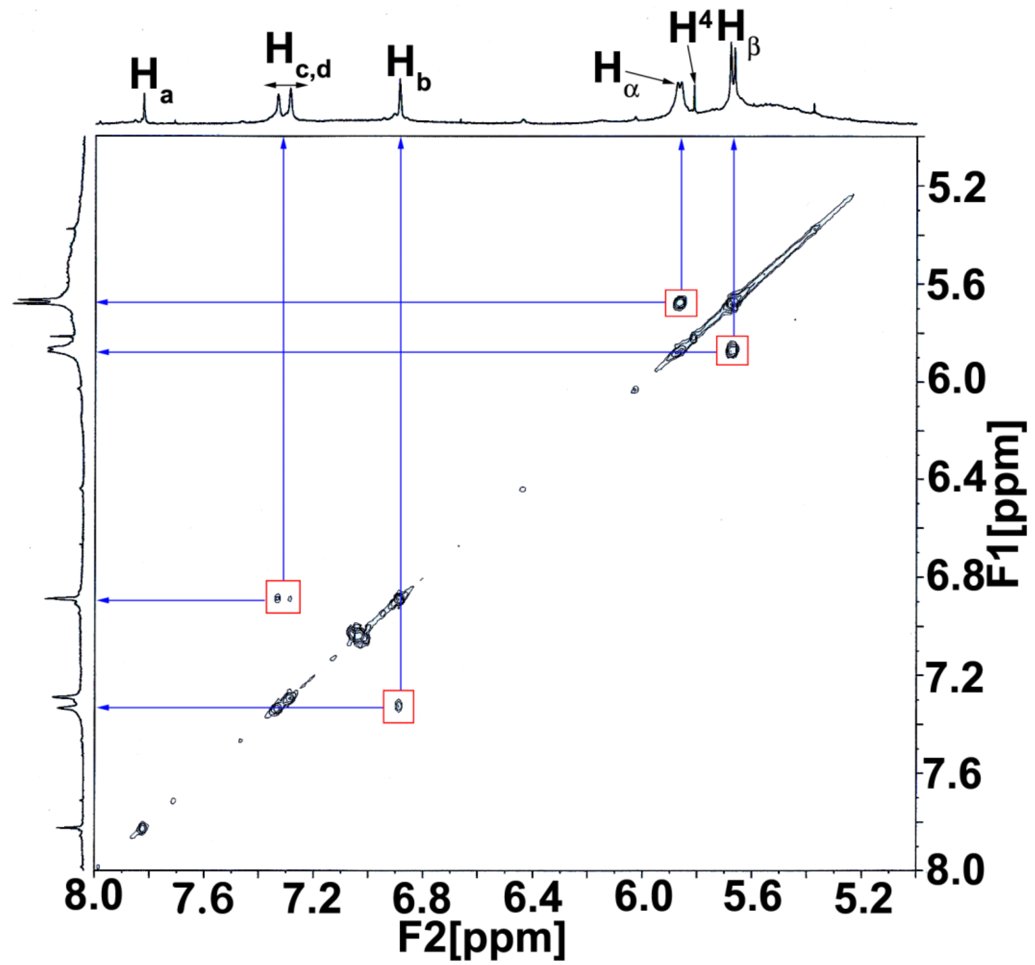

Figure S5: $^{1}H-^{1}H$ NMR spectrum of the macrocyclic cage **2c** recorded in CD_3CN.

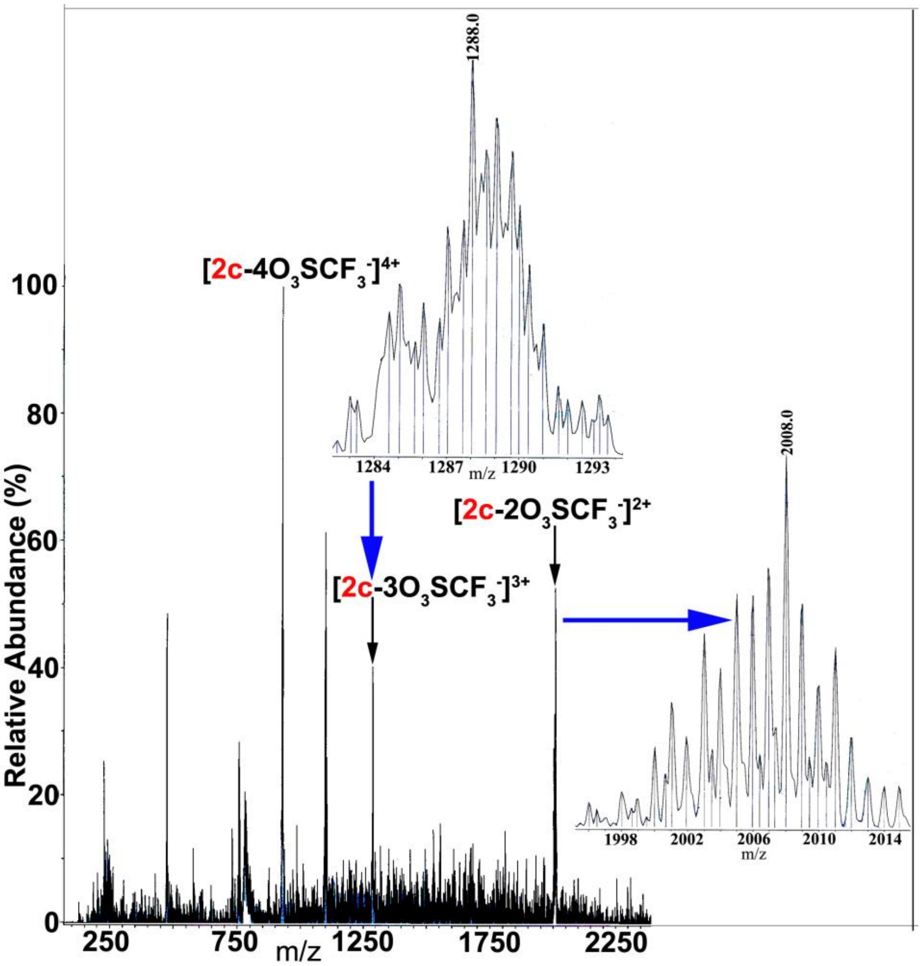

Figure S6: ESIMS spectrum of the macrocyclic cage **2c** recorded in acetonitrile. Inset: experimentally observed isotopic distribution for the [**2c** – $2O_3SCF_3^-$]$^{2+}$ and [**2c** – $3O_3SCF_3^-$]$^{3+}$ fragments.

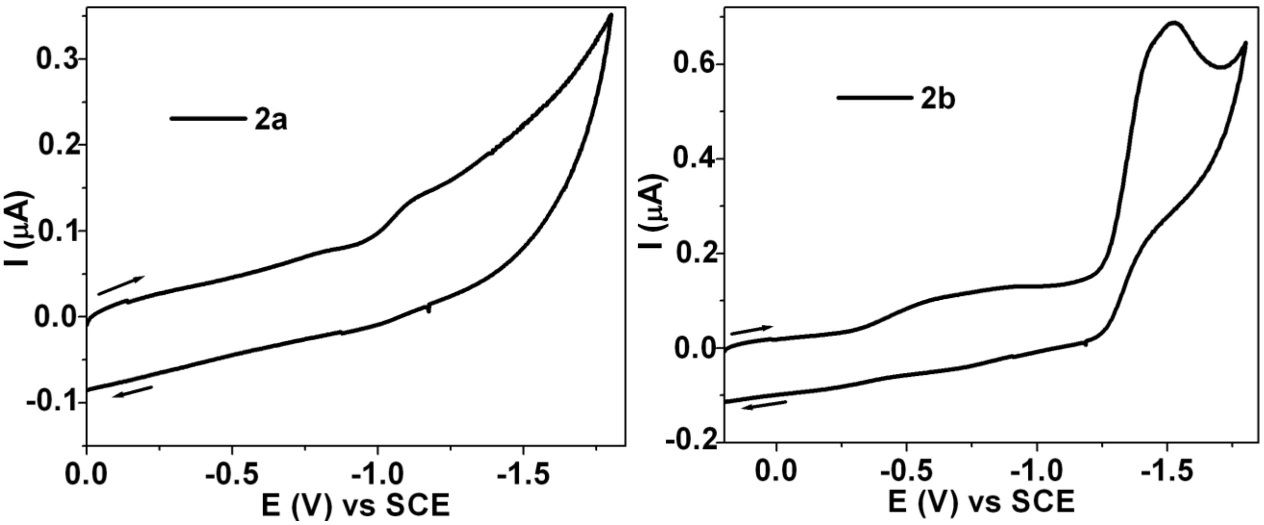

Figure S7: Cyclic voltammogram of macrocycle **2a** (left) and **2b** (right) performed in CH_2Cl_2/0.1M $(n\text{-}Bu)_4NPF_6$ with a scan rate of 100 mV s^{-1} versus SCE at 298 K.

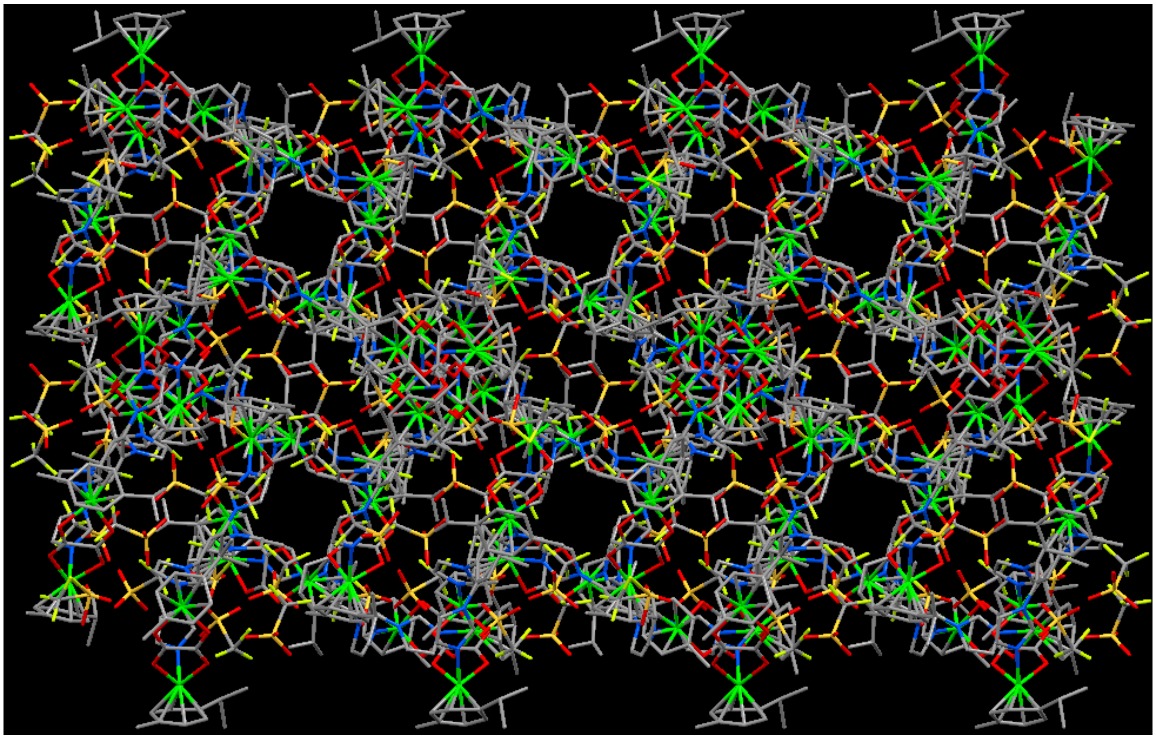

Figure S8: Solid-state packing diagram along crystallographic *c*-axis of macrocycle **2a.**

Table S1: Selected bond distances (Å) and angles (deg) for **2a**.

2a

Ru(3)-O(5)	2.117(14)	Ru(3)-N(6)	2.135(13)	Ru(3)-O(6)	2.139(11)
Ru(3)-C(25)	2.15(2)	Ru(3)-C(21)	2.16(3)	Ru(3)-C(23)	2.16(3)
Ru(3)-C(24)	2.14(4)	Ru(3)-C(22)	2.17(2)	Ru(3)-C(26)	2.17(3)
Ru(4)-C(36)	2.12(2)	Ru(4)-O(8)	2.143(10)	Ru(4)-C(35)	2.15(2)
Ru(4)-C(32)	2.161(17)	Ru(4)-N(8)	2.159(11)	Ru(4)-O(7)	2.164(13)
Ru(4)-C(33)	2.185(16)	Ru(4)-C(31)	2.22(3)	Ru(4)-C(34)	2.224(17)

O(5)-Ru(3)-N(6)	83.4(5)	O(5)-Ru(3)-O(6)	78.9(5)
N(6)-Ru(3)-O(6)	84.7(5)	O(5)-Ru(3)-C(25)	109.0(13)
N(6)-Ru(3)-C(25)	167.3(12)	O(6)-Ru(3)-C(25)	100.1(11)
O(5)-Ru(3)-C(21)	170.9(10)	N(6)-Ru(3)-C(21)	98.1(10)
O(6)-Ru(3)-C(21)	110.2(10)	C(25)-Ru(3)-C(21)	69.2(12)
O(5)-Ru(3)-C(23)	99.2(14)	N(6)-Ru(3)-C(23)	107.7(10)
O(6)-Ru(3)-C(23)	167.2(10)	C(25)-Ru(3)-C(23)	68.4(14)
C(21)-Ru(3)-C(23)	71.8(14)	O(5)-Ru(3)-C(24)	90.4(13)
N(6)-Ru(3)-C(24)	144.4(12)	O(6)-Ru(3)-C(24)	128.5(11)
C(25)-Ru(3)-C(24)	36.8(12)	C(21)-Ru(3)-C(24)	83.2(14)
C(23)-Ru(3)-C(24)	38.8(11)	O(5)-Ru(3)-C(22)	130.5(13)
N(6)-Ru(3)-C(22)	92.5(9)	O(6)-Ru(3)-C(22)	150.1(14)
C(25)-Ru(3)-C(22)	77.4(10)	C(21)-Ru(3)-C(22)	40.7(11)
C(23)-Ru(3)-C(22)	35.6(13)	C(24)-Ru(3)-C(22)	65.4(13)

O(5)-Ru(3)-C(26)	144.2(10)	N(6)-Ru(3)-C(26)	130.5(12)
O(6)-Ru(3)-C(26)	91.7(8)	C(25)-Ru(3)-C(26)	38.3(12)
C(21)-Ru(3)-C(26)	37.8(9)	C(23)-Ru(3)-C(26)	82.5(14)
C(24)-Ru(3)-C(26)	68.6(15)	C(22)-Ru(3)-C(26)	67.7(11)
C(36)-Ru(4)-O(8)	151.6(9)	C(36)-Ru(4)-C(35)	37.1(7)
O(8)-Ru(4)-C(35)	163.1(9)	C(36)-Ru(4)-C(32)	68.5(8)
O(8)-Ru(4)-C(32)	91.7(6)	C(35)-Ru(4)-C(32)	81.2(8)
C(36)-Ru(4)-N(8)	92.5(7)	O(8)-Ru(4)-N(8)	84.5(4)
C(35)-Ru(4)-N(8)	111.9(8)	C(32)-Ru(4)-N(8)	129.1(8)
C(36)-Ru(4)-O(7)	129.5(9)	O(8)-Ru(4)-O(7)	78.1(4)
C(35)-Ru(4)-O(7)	99.4(10)	C(32)-Ru(4)-O(7)	146.4(7)
N(8)-Ru(4)-O(7)	82.1(4)	C(36)-Ru(4)-C(33)	79.6(8)
O(8)-Ru(4)-C(33)	97.5(6)	C(35)-Ru(4)-C(33)	67.6(8)
C(32)-Ru(4)-C(33)	38.1(6)	N(8)-Ru(4)-C(33)	167.0(8)
O(7)-Ru(4)-C(33)	110.9(8)	C(36)-Ru(4)-C(31)	39.0(7)
O(8)-Ru(4)-C(31)	113.4(8)	C(35)-Ru(4)-C(31)	69.9(9)
C(32)-Ru(4)-C(31)	38.7(7)	N(8)-Ru(4)-C(31)	97.8(6)
O(7)-Ru(4)-C(31)	168.5(8)	C(33)-Ru(4)-C(31)	69.5(8)
C(36)-Ru(4)-C(34)	67.5(8)	O(8)-Ru(4)-C(34)	124.5(7)
C(35)-Ru(4)-C(34)	38.6(7)	C(32)-Ru(4)-C(34)	67.1(8)
N(8)-Ru(4)-C(34)	148.8(8)	O(7)-Ru(4)-C(34)	92.4(7)
